# Age group differences in psychological distress and leisure-time exercise/socioeconomic status during the COVID-19 pandemic: a cross-sectional analysis during 2020 to 2021 of a cohort study in Japan

**DOI:** 10.3389/fpubh.2023.1233942

**Published:** 2023-10-25

**Authors:** Yoshinobu Saito, Sho Nakamura, Kaname Watanabe, Hiromi Ikegami, Naoko Shinmura, Shinya Sato, Yohei Miyagi, Hiroto Narimatsu

**Affiliations:** ^1^Faculty of Sport Management, Nippon Sport Science University, Yokohama, Japan; ^2^Graduate School of Physical Education, Health and Sport Studies, Nippon Sport Science University, Tokyo, Japan; ^3^Center for Innovation Policy, Kanagawa University of Human Services, Kawasaki, Japan; ^4^Cancer Prevention and Control Division, Kanagawa Cancer Center Research Institute, Yokohama, Japan; ^5^Graduate School of Health Innovation, Kanagawa University of Human Services, Kawasaki, Japan; ^6^Department of Genetic Medicine, Kanagawa Cancer Center, Yokohama, Japan; ^7^Hygeia Communication General Incorporated Association, Kawasaki, Japan; ^8^Morphological Information Analysis Laboratory, Kanagawa Cancer Center Research Institute, Yokohama, Japan; ^9^Molecular Pathology and Genetics Division, Kanagawa Cancer Center Research Institute, Yokohama, Japan; ^10^Department of Pathology, Kanagawa Cancer Center, Yokohama, Japan

**Keywords:** COVID-19, mental health, physical activity, social environments, social disparities

## Abstract

**Objective:**

This study aimed to determine the association between psychological distress and leisure-time exercise/socioeconomic status by age group, using data from a cohort study in Japan during the COVID-19 pandemic.

**Methods:**

This cross-sectional study was conducted among participants in the ME-BYO cohort, aged 20–85 years, living or working in Kanagawa, Japan. A questionnaire was disseminated to 1,573 participants (51.7% men) between December 2020 and March 2021. The questionnaire items included psychological distress (using the 6-item Kessler Psychological Distress Scale [K6]), leisure-time exercise, and socioeconomic status. Multivariate analyses were conducted using logistic regression analysis for each age group.

**Results:**

We found that 47.4% of 20–39-year-olds, 40.6% of 40–64-year-olds, and 28.3% of 65–85-year-olds experienced psychological distress (K6: ≥5 points). For those aged 20–39 years, leisure-time exercise (odds ratio [OR] (95% confidence interval) = 0.45 (0.28–0.73)) and higher annual household income [0.53 (0.32–0.90)] were associated with less psychological distress. For those aged 40–64 years, older age was associated with less psychological distress, while full-time work [1.98 (1.05–9.71)] was associated with more psychological distress. In the 65–85-year age group, higher education and higher annual income tended to be associated with less psychological distress. For those over 40 years of age, living with other(s) was associated with reduced psychological distress.

**Conclusion:**

In the general population of Japan, not engaging in leisure-time exercise and low income affect psychological distress among young adults. Further detailed studies are needed to consider overall physical activity, job type, and work style.

## 1. Introduction

Physical activity (PA) is effective in preventing and improving non-communicable diseases and in maintaining and improving mental health (1). Moreover, several systematic reviews and meta-analyses have reported strong evidence that regular PA and exercise interventions reduce the risk and symptoms of anxiety and depression. For example, a meta-analysis of 25 randomized controlled trials of exercise interventions for patients with depression concluded that exercise is an evidence-based treatment for depression (2). Additionally, a meta-analysis of 49 prospective studies concluded that PA can prevent the onset of depression, regardless of age or region (3). Another meta-analysis suggested that engaging in PA protects against anxiety symptoms and disorders (4).

Despite this evidence, inactive lifestyles are common worldwide. The global economic burden of physical inactivity is significant. If the prevalence of physical inactivity remains unchanged, nearly 500 million new and preventable cases of non-communicable diseases and mental health problems are projected to occur between 2020 and 2030, with direct health-care costs reaching international $520 billion (5). Furthermore, people’s daily lives have been severely constrained by the coronavirus disease (COVID-19) pandemic. Lockdown and other measures to prevent COVID-19 infection forced people to reduce their range of activities, which reduced PA (6, 7) and affected mental health (8). In a meta-analysis of 20 studies from 12 countries that examined step counts before and after the COVID-19 pandemic, the percentage of studies that included participants with more than 7,000 steps per day decreased from 70% (before the pandemic) to 25% (during the confinement period). Daily step counts decreased by approximately 2,000 steps on average (7). An estimated 53.2 million (27.6%) more people worldwide had depression and 76.2 million (25.6%) more people had anxiety when compared to prior to the COVID-19 pandemic, and daily trends in infection rates and reduced human mobility (indicators of the impact of COVID-19) were associated with an increased prevalence of major depressive disorder and anxiety disorders (8). A rapid systematic review examining the association between PA and depression and anxiety during the COVID-19 pandemic found that people with longer periods of moderate-to-vigorous PA were 12–32% and 15–34% less likely to experience depression and anxiety, respectively (9). In addition, a study of university students reported that those with greater reductions in PA had worse mental health (10), and lower PA and less time spent outdoors were associated with higher anxiety and depression scores (11). Moreover, regular moderate-to-vigorous-intensity leisure-time exercise has been recommended to maintain and improve mental health during the COVID-19 pandemic (12).

In planning mental health policies, the characteristics of individuals at high risk for mental disorders should be evaluated by generation, taking into account the social environment and the social security systems of each country. In an Austrian online survey, the COVID-19 pandemic and subsequent lockdowns were particularly stressful for the young adult (<35 years of age), female, unemployed, low-income, and inactive populations (13). Studies examining the association between psychological distress and sociodemographic factors in the COVID-19 pandemic among the general population in Japan have identified the presence of a spouse (14), high annual household income (14, 15), and higher educational attainment (14, 15) as positive relevant factors. However, most of the previous studies have used web-based surveys and exhibited notable selection bias. This study was conducted on the general population in Kanagawa Prefecture, Japan, and it may be directly useful in addressing the mental health concerns of this prefecture’s residents. In addition, moderate-to-vigorous-intensity leisure-time exercise, which is effective in maintaining and improving mental health should be evaluated. In this study, we aimed to clarify the relationship between psychological distress and leisure-time exercise/socioeconomic status by age group, utilizing data from citizens who participated in a cohort study in Japan during the COVID-19 pandemic.

## 2. Methods

### 2.1. Study design and participants

This was a cross-sectional study conducted as part of the Kanagawa Prospective “ME-BYO” Cohort Study (ME-BYO cohort) in Japan (16, 17), which is one site of a collaborative genomic cohort study, namely the Japan Multi-Institutional Collaborative Cohort Study (J-MICC Study) (18). At the Kanagawa Cancer Center Research Institute (KCC), baseline recruitment began in 2016 and the baseline survey will continue through 2023. Participants in the ME-BYO cohort were aged 20–85 years and lived or worked in Kanagawa Prefecture, Japan.

The data were obtained from participants recruited from December 2020 to March 2021 from two sites: the Driver’s License Examination Center of Kanagawa Prefecture in Yokohama city and a manufacturing company located in Hiratsuka city, Kanagawa, Japan. Passers-by near the Driver’s License Examination Center of Kanagawa Prefecture were asked for voluntary cooperation after providing their informed consent. Registered residents of Kanagawa Prefecture attend this center regardless of their residence in Kanagawa; therefore, the participants were diverse and to some extent representative of the entire prefecture. At the second site, employees were sent an invitation to participate in the study along with a request for informed consent. Recruitment was conducted in conjunction with research to clarify the subclinical infection rate in the general population. Thus, persons without a history of COVID-19 were eligible. The history of infection was confirmed by self-report based on whether the participants had ever tested positive by polymerase chain reaction or antigen tests for SARS-CoV-2 (19).

A total of 1,573 participants in the ME-BYO cohort were recruited during the study period. Participants were instructed to respond to two questionnaires: (1) a baseline questionnaire for the genomic cohort study and (2) a questionnaire to clarify the subclinical infection rate in the general population (additional baseline questionnaire). Completion of both questionnaires was mandatory for participation in the study.

### 2.2. Ethical approval

All research procedures were approved by the KCC ethics committee (28KEN-36, 2020EKI-79). Written informed consent was obtained from all participants to be included in the ME-BYO cohort and to participate in the research to clarify the subclinical infection rates in the general population, respectively.

### 2.3. Measurements

The Psychological Distress Scale (K6) score, a robust non-specific psychological distress measurement tool (20, 21), is calculated from six items using a five-point Likert scale, with a total score ranging from 0 to 24; a higher score indicates more severe distress. We used a Japanese version of the scale (21) translated and validated from the original scale developed in English (20). The participants were then divided into two groups: those with high scores (poor mental health conditions: ≥ 5 points) and those with low scores (good mental health conditions: ≤ 4 points). Similarly, several previous studies have used a cut-off value of 5 points for this scale (16, 22, 23).

The frequency categories (assigned average days per week in parentheses) for moderate-to-vigorous leisure-time exercise were as follows: almost none (0), one to three times per month (0.1), one to two times per week (0.2), three to four times per week (0.5), and more than 5 times per week (0.8). The average duration categories (assigned hours per activity in parentheses) were as follows: <30 min (0.3), 30 min to <1 h (0.8), 1 to <2 h (1.5), 2 to <3 h (2.5), 3 to <4 h (3.5), and ≥ 4 h (4.5).

Leisure-time exercise was assessed by metabolic equivalents (METs) of leisure-time exercise. Participants were asked about the frequency and average duration of exercise behavior according to three broad categories of intensity (vigorous, moderate, and light). Vigorous activities, defined as those that cause a person to breathe more heavily than normal (to the extent that they cannot talk), were allocated 8.0 METs; moderate activities, defined as those that cause a person to breathe somewhat more heavily than normal (to the extent that they can still talk), were allocated 4.0 METs; and light activities, defined as those that cause a person to breathe normally (e.g., walking), were allocated 3.3 METs (24, 25). The minutes per week of leisure-time exercise were calculated using the weekly frequency and duration of leisure-time exercise. Leisure-time exercise was classified into two groups: not performed and performed.

Data on age, sex, height, weight, and living arrangement (living alone or living with other(s)) were collected as basic attributes. Age was classified into three categories based on the Japanese health examination classification for the analyses (20–39, 40–64, and 65–85 years). Socioeconomic status was determined based on the following parameters: educational attainment (up to junior college/technical school, college degree, or higher), employment type (unemployed, part-time, or full-time), and annual household income. Annual household income was categorized into two groups based on the median (≤6, >6 million yen/year [~45 thousand US dollars]).

### 2.4. Statistical analysis

Age comparisons were performed using one-way ANOVA for comparing the means (age, body mass index, K6 score: mean), the chi-squared test for comparing proportions (sex, living arrangement, leisure-time exercise: categories, educational attainment, employment type, annual household income, K6 score: cut-off value), and the Kruskal–Wallis test for comparing medians (leisure-time exercise: median). Multivariate analyses were conducted using logistic regression analysis for each age group with the K6 score as the dependent variable and age, sex, living arrangement, leisure-time exercise, educational attainment, employment type, and annual household income as independent variables. Sensitivity analyses were conducted by inputting missing data using multiple imputation methods to create ten complete datasets for separate analyses (26). Living arrangement had the lowest rate of missing values (0.6%), and employment type had the highest rate of missing values (11.5%). The significance level was set at 5%. All analyses were performed in IBM SPSS Statistics Ver. 27 (IBM Corp, Tokyo, Japan).

## 3. Results

### 3.1. Participant characteristics

Table 1 shows the characteristics of the participants. The mean (standard deviation) age of all participants was 51.2 (15.0) years. The participants aged 20–39 years had the highest rate of a K6 score ≥ 5, and the mean value was also higher. This group also had the highest number of participants who did not engage in leisure-time exercise.

**Table 1:** Participant characteristics.

	Overall (*n* = 1,573)	20–39 years (*n* = 364)	40–64 years (*n* = 919)	65–85 years (*n* = 290)	*p-*value^a^
**Age, years**					
Mean (SD)	51.2 (15.0)	30.3 (6.2)	52.8 (6.5)	72.5 (5.2)	<0.001
**Sex, *n* (%)**					
Male	813 (51.7)	178 (48.9)	457 (49.7)	178 (61.4)	0.001
Female	760 (48.3)	186 (51.1)	462 (50.3)	112 (38.6)	
**BMI**					
Mean (SD)	23.2 (3.6)	22.5 (3.7)	23.6 (3.8)	22.9 (2.9)	<0.001
**Living arrangement, *n* (%)**					
Living alone	234 (15.0)	87 (24.2)	105 (11.5)	42 (14.5)	<0.001
Living with other(s)	1,329 (85.0)	273 (75.8)	809 (88.5)	247 (85.5)	
**Leisure-time exercise**					
Not performed, *n* (%)	579 (37.6)	157 (43.7)	371 (41.2)	51 (18.2)	0.001
Performed, *n* (%)	961 (62.4)	359 (56.3)	901 (58.8)	229 (81.8)	
Min/week, median (IQR)	100.8 (33.6–273.0)	94.5 (12.6–231.0)	75.6 (25.2–231.0)	268.8 (96.6–504.0)	<0.001
**Educational attainment, *n* (%)**					
Up to junior college/technical school	848 (54.5)	180 (49.9)	511 (56.3)	157 (54.5)	0.117
College degree or higher	709 (45.5)	181 (50.1)	397 (43.7)	131 (45.5)	
**Employment type, *n* (%)**					
Unemployed	241 (17.3)	73 (21.9)	73 (8.5)	95 (47.3)	<0.001
Part-time	232 (16.7)	33 (9.9)	168 (19.6)	31 (15.4)	
Full-time	919 (66.0)	228 (68.3)	616 (71.9)	75 (37.3)	
**Annual household income, *n* (%)**					
<6 million yen	641 (42.9)	154 (45.7)	307 (34.6)	180 (66.4)	<0.001
≥6 million yen	854 (57.1)	183 (54.3)	580 (65.4)	91 (33.6)	
**K6 score**					
<5, *n* (%)	924 (60.0)	189 (52.6)	535 (59.4)	200 (71.7)	<0.001
≥5, *n* (%)	615 (40.0)	170 (47.4)	366 (40.6)	79 (28.3)	
Mean (SD)	4.4 (4.7)	5.4 (5.3)	4.5 (4.6)	3.2 (3.5)	<0.001

SD, standard deviation; BMI, body mass index; IQR, interquartile range. Sample sizes vary due to missing values.

a *p* values for comparison between age groups were calculated by one-way ANOVA, the χ^2^-test, and the Kruskal–Wallis test.

### 3.2. Relationship between psychological distress and leisure-time exercise/socioeconomic status

The association between psychological distress and leisure-time exercise/socioeconomic status is shown in Table 2. The results of the analysis, in which K6 scores ≤4 were used as the reference, showed that age (odds ratio [OR] (95% confidence interval [CI]) = 0.98 (0.97–0.99)), leisure-time exercise (performed) [0.75 (0.60–0.95)], and employment type (full-time) [1.81 (1.21–2.72)] were significantly associated with psychological distress among overall participants. For those aged 20–39 years, leisure-time exercise [0.45 (0.28–0.73)] and higher annual household income [0.53 (0.32–0.90)] were associated with less psychological distress. While those aged 40–64 years showed significant associations with age [0.97 (0.95–0.99)] and employment type [1.98 (1.05, 3.71)]. Moreover, a high odds ratio was found for annual household income (≥6 million yen) [1.33 (0.96, 1.84)], although no significant association was found. Among those aged 65–85 years, lower K6 scores tended to correlate with educational attainment (college degree or higher) and annual household income (≥6 million yen), although no significant associations were found. Although the results of the sensitivity analysis with multiple imputations showed a similar trend, living with other(s) [0.60 (0.39–0.93)] was associated with less psychological distress in the 40–64 years age group (Supplementary Table S1).

**Table 2:** Associations of leisure-time exercise and socioeconomic status with psychological distress.

	Overall			20–39 years			40–64 years			65–85 years		
Independent variables	OR	95%CI	*p*-value	OR	95%CI	*p*-value	OR	95%CI	*p*-value	OR	95%CI	*p*-value
**Age**	**0.98**	**0.97–0.99**	**<0.001**	1.02	0.97–1.06	0.493	**0.97**	**0.95–0.99**	**0.004**	1.03	0.96–1.11	0.423
**Sex**												
Male	1			1			1			1		
Female	0.88	0.68–1.14	0.326	0.65	0.40–1.08	0.094	0.94	0.66–1.34	0.739	0.97	0.43–2.22	0.950
**Living arrangement**												
Living alone	1			1			1			1		
Living with other(s)	0.75	0.54–1.04	0.085	1.27	0.70–2.31	0.426	0.65	0.41–1.04	0.071	0.54	0.20–1.43	0.215
**Leisure-time exercise**												
Not performed	1			1			1			1		
Performed	**0.75**	**0.60–0.95**	**0.019**	**0.45**	**0.28–0.73**	**0.001**	0.82	0.61–1.11	0.197	1.44	0.58–3.61	0.432
**Educational attainment**												
Up to junior college/technical school	1			1			1			1		
College degree or higher	0.82	0.64–1.05	0.111	1.62	0.96–2.73	0.070	0.51	0.73–1.00	0.051	0.53	0.26–1.10	0.090
**Employment type**												
Unemployed	1			1			1			1		
Part-time	1.25	0.89–1.77	0.202	1.06	0.52–2.17	0.881	1.31	0.72–2.41	0.380	1.35	0.56–3.28	0.505
Full-time	**1.81**	**1.21–2.72**	**0.004**	1.87	0.70–4.98	0.213	**1.98**	**1.05–3.71**	**0.034**	1.39	0.53–3.70	0.506
**Annual household income**												
<6 million yen	1			1			1			1		
≥6 million yen	0.99	0.78–1.27	0.950	**0.53**	**0.32–0.90**	**0.019**	1.33	0.96–1.84	0.084	0.47	0.20–1.08	0.075

OR, odds ratio; CI, confidence interval. Boldface indicates statistical significance (*p* < 0.05).

## 4. Discussion

In this study, approximately half of those aged 20–39 years experienced psychological distress (K6 ≥ 5 points) during the COVID-19 pandemic, which was higher than any other age group. This finding was similar to that of a web-based survey conducted in Japan during the early stages of the pandemic (14).

Among those aged 20–39 years, leisure-time exercise and higher annual household income were associated with lower psychological distress. In contrast, among those aged 40–64 years, full-time work was associated with increased psychological distress. Among those aged 65–85 years, higher education and higher annual income tended to be associated with reduced psychological distress. Sensitivity analysis showed that living with other(s) was associated with less psychological distress at ages 40–64 and 65–85. Most of the cohabitants were spouses, suggesting that the presence of close relatives may also be an important factor for psychological stability after age 40.

More than 40% of the 20–39 and 40–64-year-olds did not engage in leisure-time exercise. However, leisure-time exercise was associated with reduced psychological distress only among those aged 20–39 years. The results suggest that socioeconomic status was more strongly associated with psychological distress during the COVID-19 pandemic than was exercise among those aged 40–64 and 65–85 years. The results may have been influenced by the fact that approximately 82% of the 65–85-year-olds engaged in some leisure-time exercise. Previous studies have suggested that a high level of overall PA, including daily living activities, is a predictor of good mental health among older adults (27). In this study, analyses were conducted using a common index to examine differences between generations. However, overall PA, including activities of daily living, should also be evaluated because leisure-time increases among older adults, making it difficult to categorize it with other activities. It is also possible that COVID-19 restrictions made people more likely to exercise at home, which requires higher income levels. A study of Japanese adults showed that leisure-time exercise was less common than were other physical activities (transport and work) in lower income/education populations (28). In other words, whether socioeconomic status affects psychological distress via leisure-time exercise needs to be investigated in the future.

Most previous studies have reported that higher household income is associated with less psychological distress (14, 15, 29, 30). In the present study, higher household income was associated with less psychological distress among those aged 20–39 and 65–85 years. During the period of this study (December 2020 to March 2021), Japan was in its second state of emergency declaration (January 8, 2021 to March 21, 2021). During the declared state of emergency, workers were strongly suggested to refrain from leaving their homes and to work remotely, except when necessary. Many industries, including restaurants, fitness facilities, and leisure facilities, were closed and suffered significant losses. These factors likely affected the study participants whose psychological distress increased due to changes in income and working environment. On the other hand, a non-significant inverse association was found in the 40–64 years age group between full-time work and increased psychological distress, suggesting that the 40–64 years age group may have been affected by being in a more responsible position at work and working place infection control practices (leave-of-absence instructions, instructions for shortening business hours, and requests to avoid the working place in case of any symptoms) (31). In addition, high psychological distress among healthcare workers has been reported, and the impact of job type should also be considered (22).

Remote work is expected to become an established option for future work arrangements. However, prolonged remote work has been reported to decrease the sense of urgency to exercise, highlighting the need to build urban structures that facilitate exercise, create work systems that allow for more time to exercise (32), and promote alternatives such as digital training (33). As a future challenge, based on our results and accumulated evidence, creating an environment that facilitates exercise, especially for those who are socially vulnerable or have difficulty accessing leisure-time exercise, is crucial.

Notably, this study was conducted among a target population that was representative of the public during the COVID-19 pandemic. One limitation, however, is that recruitment was conducted in conjunction with a study to determine the rate of subclinical COVID-19 infection in the general population, which may have included participants with health concerns or fears and may have introduced a selection bias. Another limitation is that PA is a subjective assessment. In addition, because this was a cross-sectional study, causality could not be established.

## 5. Conclusion

We examined the association between psychological distress and leisure-time exercise/socioeconomic status among cohort study participants in an urban area in Japan by age group. Among young adults, leisure-time exercise and higher annual household income were associated with lower psychological distress, whereas in the 40–64 age group with greater social responsibility, employment type was associated with increased psychological distress. Among older adults, higher education and higher annual income were associated with less psychological distress. For those over 40 years of age, cohabitation was associated with reduced psychological distress. The findings suggest the need to consider mental health policies that take these factors into account for different generations. Further detailed studies are warranted to consider overall PA, job type, and work style.

## Data availability statement

The datasets presented in this article are not readily available because data cannot be shared publicly due to ethical restrictions. Requests to access the datasets should be directed to data described in the manuscript will be made available upon application and approval from the ME-BYO cohort office (contact via the corresponding author) for researchers who meet the criteria for data sharing.

## Ethics statement

The studies involving humans were approved by Kanagawa Cancer Center ethics committee. The studies were conducted in accordance with the local legislation and institutional requirements. The participants provided their written informed consent to participate in this study. Written informed consent was obtained from the individual(s) for the publication of any potentially identifiable images or data included in this article.

## Author contributions

YS conceptualized and designed the research. SN and HN designed the ME-BYO cohort. YS, SN, KW, HI, NS, SS, YM, and HN conducted the ME-BYO cohort and provided data. YS and SN were responsible for data curation. YS was data analysis and wrote the first draft of the manuscript. SN, KW, and HN were involved in the interpretation of results. All authors contributed to the article and approved the submitted version.
