# Supplementary material for: Age group differences in psychological distress and leisure-time exercise/socioeconomic status during the COVID-19 pandemic: a cross-sectional analysis during 2020 to 2021 of a cohort study in Japan
Source: Front Public Health. 2023 Oct 25;11:1233942. doi: 10.3389/fpubh.2023.1233942 (PMC10634212; doi:10.3389/fpubh.2023.1233942)
Supplement: Supplementary file 1 [file Table_1.docx]

Supplementary Material

# Supplementary Table

**Table S-1. Associations of leisure-time exercise and socioeconomic status with psychological distress by multiple imputation**

|  | **Overall** | | |  | **20–39 years** | | |  | **40–64 years** | | |  | **65–85 years** | | |
| --- | --- | --- | --- | --- | --- | --- | --- | --- | --- | --- | --- | --- | --- | --- | --- |
| Independent variables | OR | 95%CI | *P*-value |  | OR | 95%CI | *P*-value |  | OR | 95%CI | *P*-value |  | OR | 95%CI | *P*-value |
| **Age** | **0.99** | **0.98–0.99** | **<0.001** |  | 1.04 | 0.99–1.08 | 0.089 |  | **0.97** | **0.95–0.99** | **0.005** |  | 1.01 | 0.96–1.07 | 0.615 |
| **Sex** |  |  |  |  |  |  |  |  |  |  |  |  |  |  |  |
| Male | 1 |  |  |  | 1 |  |  |  | 1 |  |  |  | 1 |  |  |
| Female | 0.95 | 0.75–1.20 | 0.678 |  | 0.74 | 0.47–1.16 | 0.187 |  | 0.92 | 0.66–1.28 | 0.618 |  | 1.17 | 0.62–2.18 | 0.630 |
| **Living arrangement** |  |  |  |  |  |  |  |  |  |  |  |  |  |  |  |
| Living alone | 1 |  |  |  | 1 |  |  |  | 1 |  |  |  | 1 |  |  |
| Living with other(s) | **0.67** | **0.49–0.90** | **0.009** |  | 1.13 | 0.65–1.98 | 0.665 |  | **0.60** | **0.39–0.93** | **0.022** |  | 0.47 | 0.22–1.01 | 0.054 |
| **Leisure-time exercise** |  |  |  |  |  |  |  |  |  |  |  |  |  |  |  |
| Not performed | 1 |  |  |  | 1 |  |  |  | 1 |  |  |  | 1 |  |  |
| Performed | **0.78** | **0.62–0.97** | **0.027** |  | **0.55** | **0.36–0.86** | **0.009** |  | 0.81 | 0.61–1.08 | 0.155 |  | 1.24 | 0.59–2.62 | 0.566 |
| **Educational attainment** |  |  |  |  |  |  |  |  |  |  |  |  |  |  |  |
| Up to junior college/technical school | 1 |  |  |  | 1 |  |  |  | 1 |  |  |  | 1 |  |  |
| College degree or higher | 0.88 | 0.71–1.10 | 0.276 |  | **1.69** | **1.05–2.74** | **0.033** |  | 0.76 | 0.56–1.02 | 0.064 |  | 0.67 | 0.37–1.20 | 0.179 |
| **Employment type** |  |  |  |  |  |  |  |  |  |  |  |  |  |  |  |
| Unemployed | 1 |  |  |  | 1 |  |  |  | 1 |  |  |  | 1 |  |  |
| Part-time | 1.24 | 0.87–1.75 | 0.227 |  | 0.97 | 0.51–1.83 | 0.916 |  | 1.20 | 0.65–2.23 | 0.554 |  | 1.00 | 0.47–2.14 | 0.994 |
| Full-time | **1.67** | **1.12–2.51** | **0.013** |  | 1.53 | 0.62–3.76 | 0.358 |  | 1.72 | 0.93–3.16 | 0.083 |  | 1.30 | 0.50–3.38 | 0.585 |
| **Annual household income** |  |  |  |  |  |  |  |  |  |  |  |  |  |  |  |
| <6 million yen | 1 |  |  |  | 1 |  |  |  | 1 |  |  |  | 1 |  |  |
| ≥6 million yen | 1.01 | 0.80–1.27 | 0.918 |  | **0.57** | **0.35–0.94** | **0.027** |  | 1.27 | 0.94–1.73 | 0.126 |  | 0.61 | 0.31–1.20 | 0.153 |

OR: odds ratio, CI: confidence interval

Boldface indicates statistical significance (*P* < 0.05).
